# Emerging roles of TRIM27 in cancer and other human diseases

**DOI:** 10.3389/fcell.2022.1004429

**Published:** 2022-09-19

**Authors:** Chengpeng Yu, Dean Rao, Tiantian Wang, Jia Song, Lei Zhang, Wenjie Huang

**Affiliations:** ^1^ Hepatic Surgery Center, Tongji Hospital, Tongji Medical College, Huazhong University of Science and Technology, Wuhan, China; ^2^ Hubei Key Laboratory of Hepato-Pancreato-Biliary Diseases, Tongji Medical College, Tongji Hospital, Huazhong University of Science and Technology, Wuhan, China; ^3^ Department of Hepatobiliary Surgery, Shanxi Bethune Hospital, Shanxi Academy of Medical Sciences, Shanxi Medical University, Jinzhong, China; ^4^ Tongji Medical College, Shanxi Tongji Hospital, Huazhong University of Science and Technology, Taiyuan, China

**Keywords:** Trim27, cancer, brain diseases, autoimmune disease, ischemia-reperfusion injury

## Abstract

As a member of the TRIM protein family, TRIM27 is a RING-mediated E3 ubiquitin ligase that can mark other proteins for degradation. Its ubiquitination targets include PTEN, IκBα and p53, which allows it to regulate many signaling pathways to exert its functions under both physiological and pathological conditions, such as cell proliferation, differentiation and apoptosis. During the past decades, TRIM27 was reported to be involved in many diseases, including cancer, lupus nephritis, ischemia-reperfusion injury and Parkinson’s disease. Although the research interest in TRIM27 is increasing, there are few reviews about the diverse roles of this protein. Here, we systematically review the roles of TRIM27 in cancer and other human diseases. Firstly, we introduce the biological functions of TRIM27. Next, we focus on the roles of TRIM27 in cancer, including ovarian cancer, breast cancer and lung cancer. At the same time, we also describe the roles of TRIM27 in other human diseases, such as lupus nephritis, ischemia-reperfusion injury and Parkinson’s disease. Finally, we discuss the future directions of TRIM27 research, especially its potential roles in tumor immunity.

## Introduction

The TRIM (tripartite-motif) family of proteins, as a large family of E3 ubiquitin ligases, are characterized by an N-terminal RING finger domain, one or two B box domains (B1 box and B2 box) and a coiled-coil region with a variable C-terminus (Hatakeyama, 2017). In humans, there are approximately 80 members of the TRIM family, which are classified in subfamilies I to XI (C-I to C-XI) based on the variable C-terminus. The variable C-terminal region includes PRY domain, SPRY domain, COS domain, fibronectin type III repeat (FNIII), acid-rich region (ACID), Meprin and TRAF-homology domain (MATH), ADP-ribosylation factor family domain (ARF), filamin-type IG domain (FIL), NHL domain, PHD domain, bromodomain (BROMO), and transmembrane region (TM) (Esposito et al., 2017; Hatakeyama, 2017). Additionally, the variable domains in the C-terminal region define the specific biochemical properties of these subfamilies and confer target specificity (Esposito et al., 2017; Bhaduri and Merla, 2021). For example, PRYSPRY is found in the C-I and C-IV subfamilies, where it could mediate protein–protein interactions, particularly in immune related proteins and give them the ability to regulate immune response (Esposito et al., 2017). Additionally, PHD and Bromodomain, contained in C-V subfamily, plays an important role in chromatin biology and transcriptional regulation and make them obtain the ability to regulate the expression of downstream genes (Nisole et al., 2005). Meantime, C-I, C-II and C-III subfamilies contain COS domain and exert a vital role in microtubule binding (Baldini et al., 2020). The NHL domain, found in the C-VII subfamily, plays an important role in protein–protein and protein-RNA interactions (Bawa et al., 2021).

TRIM27 (tripartite motif-containing 27) was firstly identified as a Rfp/Ret fusion protein, with a vital role in the full transforming activity of Rfp/Ret (Hasegawa et al., 1996). As a member of the TRIM family of proteins, TRIM27 can act as a RING-mediated E3 ubiquitin ligase to induce the ubiquitination of other proteins, such as PTEN, RIP1 and JAK1 to regulate signaling pathways (Zurek et al., 2012; Lee et al., 2013; Zaman et al., 2013; Conwell et al., 2015; Nie et al., 2016; Zhuang et al., 2016; Zhang H. X. et al., 2018). Meantime, TRIM27 belongs to C-IV subfamily and contains PRYSPRY, which could interact with immune related proteins and involved in immune response (Esposito et al., 2017). Additionally, TRIM27 was also found to play vital roles in the cell proliferation, differentiation and apoptosis (Gillot et al., 2009; Yao et al., 2020; Hao et al., 2021). Many studies demonstrated that TRIM27 might contribute to the progression of cancer, ischemia-reperfusion injury, cardiac hypertrophy and brain diseases (Gillot et al., 2009; Zaman et al., 2013; Liu et al., 2014; Conwell et al., 2015; Zheng et al., 2015; Nie et al., 2016; Zhuang et al., 2016; Zhuang et al., 2017; Li Y. et al., 2021; Yang et al., 2022). However, there are few reviews about it. Hence, we make a review about the roles of TRIM27 in cancer and other human diseases. Firstly, the biological features of TRIM27 were described. Next, we focus on the roles of TRIM27 in cancer and other human diseases. Finally, we provide the future directions of TRIM27 research, especially the discussion about its potential effect in tumor immunity.

## The structure and functions of TRIM27

**FIGURE 1:**
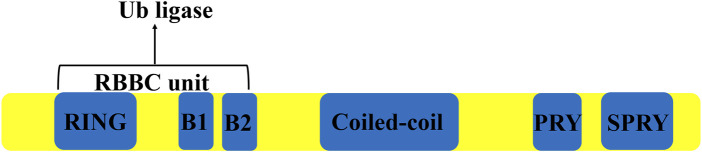
The structure of TRIM27. TRIM 27 contains a Ring domain, two boxes, a coiled-coil domain and a PRYSPRY domain.

TRIM27 is a 58 kDa protein containing 533 amino acids, encoded by the *trim27* gene on chromosome 6 in humans, consisting of six introns and seven exons spanning a length of 2,963 bps (Takahashi and Cooper, 1987). This protein contains three zinc-binding domains, a RING domain, two Box domains and a coiled-coil domain (Zoumpoulidou et al., 2012) (Figure 1). TRIM27 was found to be highly expressed in the mouse thymus, spleen and hematopoietic compartment cells (Tezel et al., 1999; Rajsbaum et al., 2008). Similar to many other TRIM proteins, the expression of TRIM27 is regulated by type I IFNs (Rajsbaum et al., 2008; Carthagena et al., 2009). However, as a member of TRIM family of proteins, TRIM27 could act as a RING-mediated E3 ubiquitin ligase to induce the ubiquitination of other proteins (Yang et al., 2022). At the same time, TRIM27 can interact with the enhancer of the polycomb protein gene to inhibit its expression (Zoumpoulidou et al., 2012).

## Signaling pathways related to TRIM27

### TRIM27 on PI3K/AKT signaling

PI3K/AKT signaling pathway is a classical signal transduction pathway that exerts an important role in cellular growth, proliferation, differentiation and apoptosis (Fresno Vara et al., 2004). In this pathway, growth factors (GFs), such as EGF, VEGFA and FGR19, could activate PI3K, which then recruits and activates AKT. The activation of AKT could exert its function by regulating its downstream substrate, such as TSC2, BAD, and MDM2 et al. (Porta et al., 2014). However, PTEN could dephosphorylate PIP3 to release PIP2, whereby the decrease of PIP3 level could inhibit the activation of PI3K/AKT signaling (Song et al., 2012). James et al. reported that TRIM27 could interact with PTEN and lead to the atypical polyubiquitinations of PTEN. However, these ubiquitinations didn’t affect the protein level of PTEN, but rather attenuated the phosphatase activity of PTEN, thus decreasing its ability to regulate PI3K/AKT signaling (Lee et al., 2013). Accordingly, TRIM27 could promote PI3K/AKT signaling by interacting with and decreasing the phosphatase activity of PTEN.

### TRIM27 on Wnt/β-catenin signaling

Wnt/β-catenin signaling pathway is a highly conserved pathway in biological evolution (Yu et al., 2021). Under normal physiological conditions, *β*- Catenin is an integral E-cadherin and acts as an intercellular adhesion adaptor protein and a transcriptional cofactors. In the absence of Wnt ligands, adenomatous polyposis coli (APC), AXIN1, casein kinase 1 (CK1) and glycogen synthase kinase 3β (GSK3β) complex phosphorylates *β*-catenin leads to its ubiquitination and subsequent proteasomal degradation to maintain a low protein level of cytosolic *β*-catenin (Liu J. et al., 2022). When Wnt ligands interact with the Frizzled receptors, AXIN1 and GSK3β are recruited to the plasma membrane by phosphorylated Dvl/Dsh, which protects *β*-catenin from being phosphorylated and degraded (Nusse and Clevers, 2017). Accumulated cytosolic *β*-catenin could translocate into the nucleus and interact with the TCF/LEF complex to transactivate downstream genes (Nusse and Clevers, 2017). SIX3 is a member of the sine oculis homeobox transcription factor family, which could inhibit the expression of both Wnt1 and Wnt8b to negatively regulate the activation of Wnt/β-catenin signaling (Kumar et al., 2010). TRIM 27 was reported to interact with and ubiquitinates SIX3, whose subsequent proteasomal degradation could promote the activation of Wnt/β-catenin signaling (Liu et al., 2020).

### TRIM27 on NF-κB signaling

NF-κB is a eukaryotic transcription factor that is involved in the control of cellular growth and differentiation, immune response, inflammation and tumorigenesis (Yu et al., 2020). Under the physiological condition, IκBs, such as IκBα、IκBβ、IκBγ and IκBε, could interact with and prevent NF-κB from translocating into the nucleus to stimulate the expression of downstream genes by covering the nuclear localization signal (NLS) of NF-κB (Panahi et al., 2016). Under various stimuli, IκBs could be phosphorylated, whose subsequent proteasomal degradation could uncover the NLS of NF-κB and make it translocate into the nucleus to promote the expression of downstream genes (Yamamoto and Gaynor, 2001). Additionally, TRIM27 was reported to interact with IκBα and lead to the ubiquitination of IκBα (Xiao et al., 2021). Meantime, TRIM27 could promote the activation of NF-κB signaling. Taken together, TRIM27 might regulate NF-κB signaling by ubiquitinating IκBα (Xiao et al., 2021).

### TRIM27 on JAK/STAT3 signaling

The JAK-STAT3 signaling pathway is a cytokine-stimulated signal transduction pathway, containing receptor tyrosine kinases (RTKs), Janus kinases (JAKs), and signal transducer and activator of transcription 3 (STAT3) (Xu et al., 2022). JAKs includes JAK1, JAK2, JAK3 and TYK2 family members (Xu et al., 2022). In this signaling pathway, Various cytokines, such as EGF, HGF, IL-6 and TGFβ could activate PKTs, which recruits and phosphorylates JAKs(Jin, 2020). The activation of JAKs could phosphorylate STATA3, which then translocate into the nucleus and promote the expression of downstream genes (Jin, 2020). TRIM27 was reported to interact with JAK1 and STAT3 and essential for JAK1–STAT3 complex formation (Zhang H. X. et al., 2018). Additionally, TRIM27 could promote the activation of JAK/STAT3 signaling by enhancing the association between JAK1 and STAT3 (Zhang H. X. et al., 2018).

## Role of TRIM27 in cancer

**FIGURE 2:**
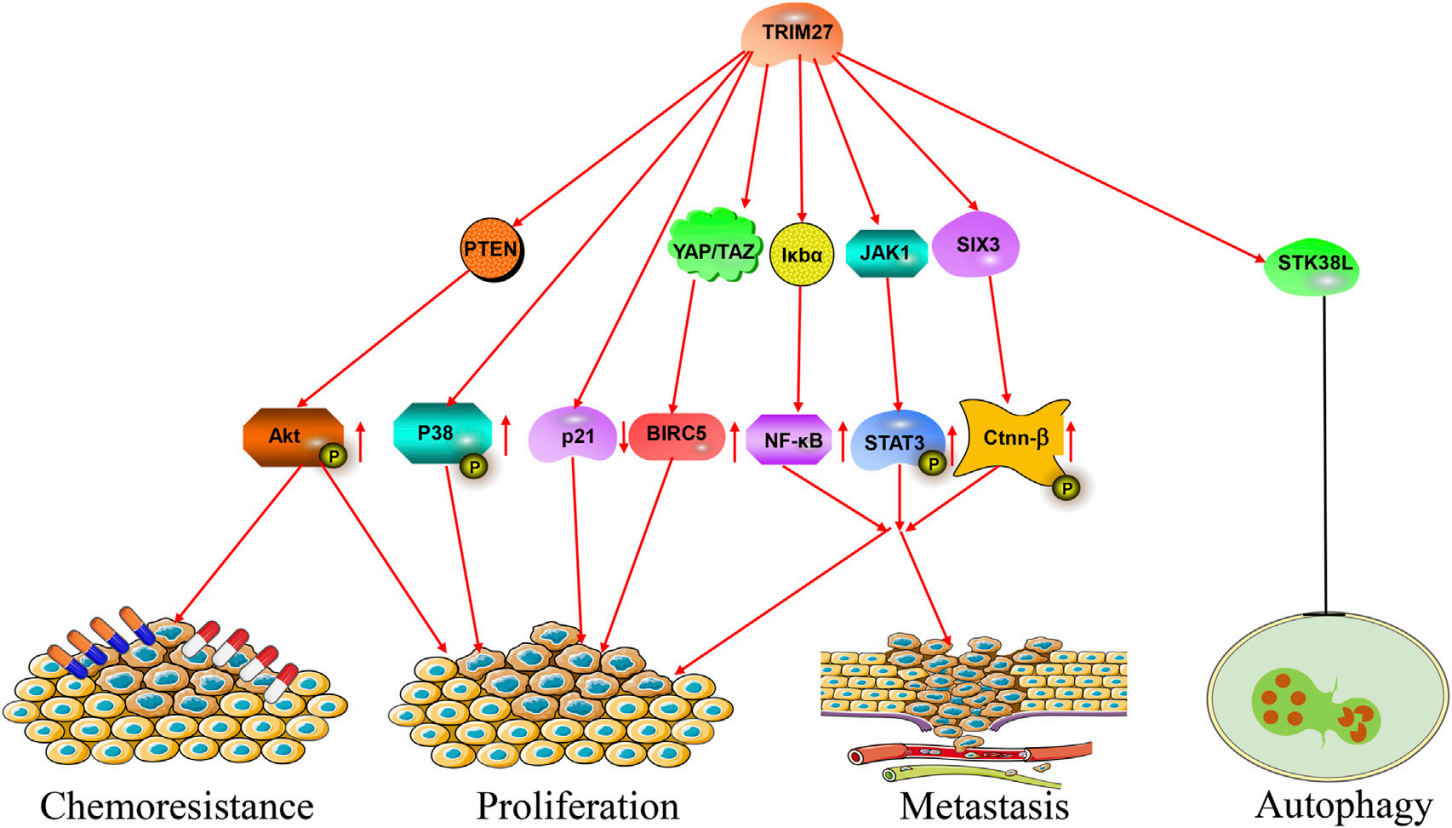
The roles of TRIM27 in cancer. TRIM27 exerts multiple effects, such as proliferation, metastasis, autophagy and chemoresistance via many signaling pathways.

During the past decades, numerous studies showed that TRIM27 was abnormally expressed in many kinds of cancer. For example, TRIM27 was highly expressed in hepatocellular carcinoma, non-small-cell lung cancer (NSCLC), ovarian cancer and breast cancer (Ma et al., 2016; Liu et al., 2020; Xing et al., 2020; Sakamoto et al., 2022) (Figure 2; Table 1). High TRIM27 expression in these kinds of cancer was associated with worse clinicopathological features and a poor prognosis (Ma et al., 2016; Zhang H. X. et al., 2018; Liu et al., 2020; Xing et al., 2020; Sakamoto et al., 2022). The details were as follows.

**TABLE 1:** The roles of TRIM27 in cancer.

Cancer type	The expression of TRIM27	Functions of TRIM27	Related signaling pathways	References
Ovarian cancer	High expression	Promote cell proliferation and chemoresistance	PI3K-AKT signaling	Horio et al. (2012), Ma et al. (2016), Jiang et al. (2019)
Breast cancer	High expression	Promote tumorigenesis; Inhibit autophagy	P21 signaling; ULK1 signaling	Xing et al. (2020), Yang et al. (2022)
Lung cancer	High expression	Promote proliferation and metastasis	Wnt/β-catenin signaling	Ji et al. (2020), Liu et al. (2020)
Colorectal cancer	High expression	Promote tumorigenesis	PI3K-AKT signaling; IL6-STAT3 signaling	Zhang et al. (2018a), Zhang et al. (2018b)
Hepatocellular carcinoma	High expression	Promote proliferation and metastasis	STAT3 signaling	Mao et al. (2021), Sakamoto et al. (2022)
Gastric cancer	High expression	Promote cell proliferation and chemoresistance	Hippo-BIRC5 signaling	Yao et al. (2020)
Renal cell carcinoma	High expression	Promote proliferation and inhibit apoptosis	NF-κB signaling	Xiao et al. (2021)
Skin cancer	—	Inhibit tumorigenesis	—	Zoumpoulidou et al. (2012)

Ovarian cancer is the eighth most common cancer in women (Lheureux et al., 2019; Stewart et al., 2019). In 2022, there were about 21,000 new cases of ovarian cancer in the United States (Siegel et al., 2021). In ovarian cancer, the expression of TRIM27 was significantly related to metastasis and FIGO stag (Ma et al., 2016). At the same time, downregulation of TRIM27 expression inhibited the proliferation of ovarian cancer cells *in vivo* and *in vitro* by upregulating the phosphorylation of p38 and downregulating the phosphorylation of AKT, (Ma et al., 2016). However, the exact mechanism that TRIM27 upregulates the phosphorylation of p38 and downregulates the phosphorylation of AKT needs further investigations. In addition, other studies demonstrated that TRIM27 could enhance cellular proliferation and chemoresistance by activating PI3K-AKT signaling (Horio et al., 2012; Jiang et al., 2019), further validating the above results.

Breast cancer is the most frequently diagnosed cancer in women and ranks second among causes for cancer related deaths in women (Harbeck and Gnant, 2017; Nardin et al., 2020). TRIM27 was found to inhibit the apoptosis and senescence of cancer cells in breast cancer (Xing et al., 2020). Meantime, the overexpression of TRIM27 could enhance cellular viability and tumor growth and attenuate the anti-cancer effects of Tamoxifen (Xing et al., 2020). Additionally, TRIM27 could mediate these above effects by inducing the ubiquitination and degradation of p21 (Xing et al., 2020). Autophagy was found to stimulate the progression of advanced cancer by promoting drug resistance and immune escape (Amaravadi et al., 2019). Yang et al. demonstrated that TRIM27 could promote the tumorigenesis of breast cancer by cooperating with STK38L to inhibit Unc-51-like kinase 1 (ULK1)-induced autophagy, (Yang et al., 2022), where ULK1 is a cytoplasmic kinase that can interact with the autophagy-related gene 13 (ATG13), and focal adhesion kinase interacting protein 200 kDa (FIP200) to trigger the initiation of autophagy (Amaravadi et al., 2019).

Lung cancer is a malignant tumor originating from the bronchial mucosa or glands of the lungs (Hirsch et al., 2017; Bray et al., 2018).Non-small-cell lung carcinoma (NSCLC) is the most common type of lung cancer (Fois et al., 2021).In NSCLC, TRIM27 was found to promote the cell proliferation and metastasis *in vivo* and *vitro* by interacting with SIX3 and promoting its degradation to activate Wnt/β-catenin signaling (Liu et al., 2020). At the same time, another study demonstrated that smoking could change the methylation of the *trim27* gene, whose methylation level was associated with the overall survival of NSCLC patients (Ji et al., 2020).

Colorectal cancer (CRC) is a complex and heterogeneous carcinoma tightly associated to dietary and lifestyle factors, and increasing studies have reported that genetic alterations and epigenetic dysregulation contributed to CRC (Bhandari et al., 2017; Li J. et al., 2021). Zhang et al. demonstrated that the overexpression of TRIM27 promoted tumor growth and metastasis *in vivo* and *vitro* in CRC (Zhang Y. et al., 2018). Meantime, TRIM27 could promote the epithelial-mesenchymal transition (EMT) of CRC cells by activating PI3K-AKT signaling (Zhang Y. et al., 2018). At the same time, another study demonstrated that TRIM27 could induce colitis to promote the tumorigenesis of colitis-associated cancer by recruiting gp130 and JAK1 to activate the IL6-STAT3 signaling pathway (Zhang H. X. et al., 2018).

Hepatocellular carcinoma (HCC) is the most common gastrointestinal neoplasm, and is responsible for 500,000–600,000 deaths annually (Forner et al., 2018; Kulik and El-Serag, 2019). In HCC, TRIM27 was found to promote cell proliferation and metastasis of HCC cell lines *in vitro* by activating STAT3 (Sakamoto et al., 2022). Another study demonstrated that the knockdown of Circ_0091579 inhibited the proliferation, migration of HCC cells by suppressing cell cycle progression and promoting epithelial-mesenchymal transition (EMT). As a targeted molecule of Circ_0091579, MiR-136-5p could overturn its effects. Additionally, MiR-136-5p interacted with the 3′ untranslated region (3′UTR) of TRIM27 and decreased the expression of TRIM27. At the same time, the overexpression of TRIM27 largely attenuated the influence of miR-136-5p in HCC cells. To sum up, Circ_0091579 could promote the proliferation and migration of HCC cells *via* the miR-136-5p/TRIM27 axis (Mao et al., 2021).

Gastric cancer is a malignant tumor originating from the gastric mucosal epithelium, accounting for 738,000 deaths annually (Smyth et al., 2020; Siegel et al., 2021). Yao et al. reported that TRIM27 knockdown could suppress cell proliferation and promote cell apoptosis in gastric cancer (Yao et al., 2020). 5-Fluorouracil, a broad-spectrum chemotherapeutic agent, could block DNA replication to inhibit tumor growth. Moreover, the knockdown of TRIM27 increased the sensitivity of gastric cancer cells to 5-fluorouracil treatment (Yao et al., 2020). Additionally, it was revealed that TRIM27 could mediate the above effects by activating the Hippo-BIRC5 pathway (Yao et al., 2020).

Renal cell carcinoma is the eighth most common cancer in the United States (Bray et al., 2018). In 2018, approximately 400,000 patients were diagnosed with renal cell carcinoma (Jonasch et al., 2014; Bray et al., 2018). TRIM27 was reported to promote the tumor growth of RCC cell lines *in vivo* and *vitro* (Xiao et al., 2021). Furthermore, the expression of TRIM27 expression was positively related to NF-κB expression in RCC patients and blocking NF-κB pathway overturned the TRIM27-mediated effects.(Xiao et al., 2021). Additionally, TRIM27 could bind to Iκbα, an inhibitor of NF-κB, to promote its ubiquitination, which led to the activation of NF-κB pathway (Xiao et al., 2021). Taken together, TRIM27 might regulate NF-κB signaling to promote the growth of human renal cancer cells.

Skin cancers are the most common solid cancers in Caucasian populations, which lack strong pigment protection (Brunssen et al., 2017; Leiter et al., 2020). Zoumpoulidou et al. (2012) reported that the knockdown of TRIM27 could attenuate the chemically induced development of skin cancer in a mouse model. Retinoblastoma protein (Rb) is a negative regulator of the cell cycle and exerts a vital role in cellular senescence, which limits oncogenic transformation (Salama et al., 2014). Meantime, another study demonstrated that TRIM27 overexpression could reduce RB protein–driven senescence in human cells (Krutzfeldt et al., 2005). Additionally, the loss of TRIM27 resulted in excessive senescence in response to replicative as well as oncogene-associated stress (Zoumpoulidou et al., 2012). Accordingly, TRIM27 might decrease senescence to contribute to the progression of skin cancer *via* RB pathway.

## The roles of TRIM27 in other human diseases

### TRIM27 in the antiviral immune response

In the process of fighting against viruses, pattern recognition receptors (PRRs) sense viral nucleic acids and trigger downstream signaling pathways, resulting in the production of type I interferons (IFNs) and other proinflammatory cytokines (Lester and Li, 2014; Li B. et al., 2020). The production of type I IFN plays a vital role in the process of fighting against viruses, but excessive production of type I IFN can result in the autoimmune damage (Lester and Li, 2014). Conversely, a deficiency in the production of type I IFN can result in failure to contain the infection (Li B. et al., 2020). However, the mechanisms safeguarding the balance of type I IFN production remain unclear. Upon viral infection, pattern recognition receptors (PRRs) recognize viruses and trigger TBK1 activation *via* the key adaptors TRIF, MAVS, or STING, which then activates the transcription factor IRF3 to induce type I IFN production (O'Neill et al., 2013). Additionally, TRIM27 was reported to interact with and ubiquitinate TBK1 to induce its proteasomal degradation and thereby negatively regulate the production of type I IFN(Zheng et al., 2015; Zheng et al., 2016; Cai et al., 2018). Type I IFN can induce a decrease of miRNA-27a, which targets TRIM27 and decreases its expression (Zheng et al., 2015; Zheng et al., 2016; Cai et al., 2018). These interactions form a negative feedback loop that tightly controls the balance of type I IFN production. Notably, TRIM27 was also found to be induced by hepatitis C virus (HCV) infection (Zheng et al., 2019). The upregulation of TRIM27 can in turn promote the replication of HCV by blocking the type I IFN response (Zheng et al., 2019). At the same time, TRIM27 was found to inhibit the activation of mast cells by interacting with PI3KC2β to induce its degradation (Srivastava et al., 2012). Similarly, TRIM27 was also reported to negatively regulate CD4^+^ T cells by interacting with PI3KC2β to induce its degradation (Cai et al., 2011). However, TRIM27 was reported to inhibit the survival of mycobacteria in macrophages by enhancing innate immune responses to mycobacterial infection (Wang et al., 2016). These studies indicate that TRIM27 might play dual roles in the immune response by regulating different pathways, such as IFN and AKT signaling.

### TRIM27 in ischemia-reperfusion injury

Ischemia-reperfusion injury (IRI) is tissue damage that occurs when blood supply returns after a period of ischemia (Kalogeris et al., 2012; Zang et al., 2020). The absence of oxygen and nutrients from blood during the ischemic period creates conditions in which the restoration of circulation results in inflammation and oxidative damage (Kalogeris et al., 2012). TRIM27 was reported to be downregulated in liver tissue from liver transplantation patients. Furthermore, TRIM27 was found to attenuate liver ischemia/reperfusion injury in mice by interacting with TAB2/3 to induce its degradation and inhibit TAK1-JNK/p38 signaling (Chen et al., 2021). At the same time, TRIM27 was found to attenuate cardiac ischemia-reperfusion injury in mice by interacting with p53 and enhancing its ubiquitination (Li Y. et al., 2021). In addition, TRIM27 was reported to protect against acute kidney injury in mice by reducing inflammation and apoptosis (Li X. K. et al., 2020).

### TRIM27 in lupus nephritis

Lupus nephritis is one of the most common complications of systemic lupus erythematosus, and can gradually lead to end-stage renal disease (Lech and Anders, 2013; Almaani et al., 2017). Destruction of the glomerular filtration barrier is the most typical pathological feature of lupus nephritis (Lech and Anders, 2013). TRIM27 was reported to be highly expressed in the glomerular endothelial cells of patients with lupus nephritis, and TRIM27 knockdown could attenuate glomerular endothelial cell injury by regulating the FoxO1 signaling pathway (Liu et al., 2021). At the same time, another study reported that knockdown of TRIM27 could inhibit the proliferation of mesangial cells in lupus nephritis *via* the FoxO1 pathway (Liu et al., 2019). These findings indicate that TRIM27 contributes to the progression of lupus nephritis *via* multiple effects.

### TRIM27 in cardiac hypertrophy

The increase of myocyte size, as an adaptive response to the overload of cardiac wall stress, is defined as cardiac hypertrophy (Nakamura and Sadoshima, 2018; Zhu et al., 2019). In spite of significant research interest, the exact molecular mechanisms of cardiac hypertrophy are not clearly understood (Zhu et al., 2019). Chen et al. (2022) reported that TRIM27 was upregulated in the transverse aortic constriction (TAC) group compared to the sham operation (Sham) group, and TRIM27 knockdown could attenuate cardiac hypertrophy *in vitro* and *in vivo*. Mechanistically, TRIM27 was found to activate Akt/mTOR signaling by interacting with PTEN (Lee et al., 2013; Chen et al., 2022). TRIM 27 therefore contributes to cardiac hypertrophy by activating the PTEN/Akt/mTOR axis.

### TRIM27 in Parkinson’s disease

Parkinson’s disease (PD) is a neurodegenerative disorder characterized by slowness of movement, muscle rigidity, resting tremor, and postural instability (Kalia and Lang, 2015; Tolosa et al., 2021). Pathologically, PD is characterized by the death of dopaminergic neurons in the basal ganglia (Kalia and Lang, 2015). TRIM27 was reported to be upregulated in PBMC from PD patients compared to healthy controls, and Knockdown of TRIM27 could protect dopaminergic neurons by inhibiting apoptosis *in vitro* and *in vivo* (Liu et al., 2014). Taken together, these findings indicate that TRIM27 might play a role in the progression of PD.

### TRIM27 in epilepsy

Epilepsy is a chronic neurological disorder, in which the abnormal discharge of neurons leads to transient brain dysfunction (Pitkanen et al., 2016; Thijs et al., 2019). Glutamate-mediated neurotoxicity plays an important role in epilepsy (Tobaben et al., 2011). Hao et al. (2021) reported that TRIM27 was upregulated in patients, and glutamate treatment could induce the upregulation of TRIM27 in HT22 cells. The upregulated TRIM27 could in turn enhance glutamate-induced apoptosis and inflammation by interacting with PPARγ and leading to its degradation (Hao et al., 2021). These findings imply that TRIM27 might be involved in the progression of epilepsy.

### TRIM27 in psoriasis

Psoriasis is a chronic autoimmune disease of the skin and joints, whose classical symptoms include salmon-pink plaques in persons with white skin or grey plaques in persons with dark skin (Boehncke and Schon, 2015; Griffiths et al., 2021). Miao et al. reported that TRIM27 was upregulated in psoriatic skin, and TRIM27 knockdown could inhibit the IL-6-induced proliferation of HaCaT cells (Miao et al., 2020). Mechanistically, TRIM27 was found to interact with PIAS3 and induce its degradation to block IL-6/STAT3 signaling (Miao et al., 2020). This study indicates that TRIM27 may be involved in the development of psoriasis.

### TRIM27 in Crohn’s disease

Crohn’s disease is a type of inflammatory bowel disease (IBD) that may affect any segment of the gastrointestinal tract (Rimola et al., 2022). The clinical manifestations of this disease are abdominal pain, diarrhea, intestinal obstruction, accompanied by fever, nutritional disorders and other extraintestinal manifestations (Liu D. et al., 2022). Additionally, the polymorphisms of NOD2 (nucleotide-binding oligomerization domain containing two) were found to be with susceptibility to Crohn’s disease. NOD2 deficiency could result in dysregulated immune responses to gut bacteria to contribute to the progression of Crohn’s disease (Fritz et al., 2011). Zurek et al. (2012) reported that TRIM27 was highly expressed in Crohn’s disease patients. Additionally, TRIM27 could interact with NOD2 and make it ubiquitinated with K48-linked ubiquitin chains followed by proteasomal degradation (Zurek et al., 2012). Accordingly, TRIM27 might affect NOD2-mediated proinflammatory responses to promote the progression of Crohn’s disease.

## Conclusion and perspectives

In this paper, we systematically reviewed the roles of TRIM27 in cancer and other human diseases, such as ischemia-reperfusion injury, lupus nephritis and cardiac hypertrophy (Liu et al., 2019; Li Y. et al., 2021; Chen et al., 2022). In all the available studies on the roles of TRIM27 in cancer, TRIM27 was reported to play an oncogenic role, with no studies indicating a tumor suppressor role. By contrast, many members of the TRIM family were found to exert dual roles in the development of cancer (Hatakeyama, 2017; Jaworska et al., 2020). For example, TRIM33 acts as a tumor enhancer in some cancers, while playing a tumor suppressor role in other cancers (Yu et al., 2019). At the same time, the mRNA level of TRIM27 was found to be downregulated in cervical squamous cell carcinoma and acute myeloid leukemia in the TCGA database (Tang et al., 2017). Whether TRIM27 exerts a tumor suppressor role in these two cancers needs further investigation.

TRIM27 has been reported to promote tumorigenesis *via* multiple effects, such as promoting tumor proliferation and metastasis, inducing chemoresistance, and inhibiting autophagy (Liu et al., 2020; Yao et al., 2020; Yang et al., 2022). However, there is a lack of studies investigating the effect of TRIM27 on tumor immunity. TRIM27 was reported to play vital roles in the innate immune response, such as inhibiting the production of type I IFN and inhibiting the activation of mast cells (Srivastava et al., 2012; Zheng et al., 2016). Notably, the innate immune response plays an important role in cancer immune escape (Vesely et al., 2011; Gajewski et al., 2013). For example, NK cells can inhibit tumor proliferation by directly killing tumor cells (Vesely et al., 2011). Hence, it is urgent to explore the effects of TRIM27 on the tumor immune response, as it might become a new immunotherapy target.
